# Post-sleeve gastrectomy weight loss: Role of botulinum toxin and semaglutide injections

**DOI:** 10.1055/a-2793-9945

**Published:** 2026-02-23

**Authors:** Zhengqi Li, Shaohan Zhang, Yuntao Nie, Pengpeng Wang, Baoyin Liu, Biao Zhou, Nianrong Zhang, Siqi wang, Sai Chou, Lei Zhang, Zhe wang, Hua Meng

**Affiliations:** 1 36635 Department of General Surgery & Obesity and Metabolic Disease Center, China-Japan Friendship Hospital, Beijing, China; 2 The Third Clinical School of Medicine, Capital Medical University, Beijing, China; 3 Department of Oncology, Sinopharm Tongmei General Hospital, Beijing, China

**Keywords:** Endoscopy Upper GI Tract, Dilation, injection, stenting, Laparoscopy, GI surgery

## Abstract

**Background and study aims:**

Comparison of the therapeutic effects of endoscopic botulinum toxin (BTX) injection and semaglutide (SME) injection in treatment of patients with obesity and weight regain after laparoscopic sleeve gastrectomy (LSG).

**Patients and methods:**

The injection method for botulinum toxin is to dilute 300 U of type A botulinum toxin with 20 mL of physiological saline. Injections were made in a grid pattern throughout the antrum and body (20 injection points total), with 1 mL of drug injected at every point. The application method for semaglutide injection is to start at 0.25 mg once a week and increase by 0.25 mg every 3 weeks until the patient can tolerate the maximum dose (1.0 mg qw), for a total of 6 months.

**Results:**

The weight loss effect of the SME group was significantly better than that of the LSG+SME group because the SME group achieved significantly greater weight loss at 2 months than the LSG+SME group. No significant difference was seen between any other pair of groups.

**Conclusions:**

Endoscopic botulinum toxin injection can be a safe and effective treatment for weight regain after LSG.

## Introduction

As living standards improve, obesity has become a global health concern. In 2015, 107.7 million children and 603.7 million adults worldwide were afflicted with obesity 1 , a figure that has continued to rise. Research indicates a strong association between obesity and development of cardiovascular diseases, chronic kidney disease, and numerous types of cancer 2 3 4 .

As prevalence of severe obesity continues to grow, weight loss surgery is increasingly entering the public consciousness. However, these surgeries are inherently invasive and carry risk of prompting a range of postoperative issues, including peritonitis, fistulas, and bleeding 5 . In contrast, the latest generation of weight loss medications offers a milder approach. Semaglutide (SME), a powerful and sustained glucagon-like peptide-1 (GLP-1) analog, exhibits not only effective weight loss capabilities but also provides cardioprotection. In addition, it may serve as an adjunctive therapy following certain bariatric procedures, making it a promising candidate with significant development potential 6 7 8 . As semaglutide research advanced, it revealed gastrointestinal side effects, particularly pancreatitis and nausea/vomiting. There is also a hint that GLP-1 use might cause acute kidney injury 9 and raise risk of thyroid cancers, including medullary types, although the reasons are unclear 10 . These adverse effects are rare but challenging to predict and prevent 11 .

Botulinum toxin (BTX), a potent neurotoxin produced by the bacterium *Clostridium botulinum* , is widely used to address a variety of medical issues, including obesity. By inhibiting release of acetylcholine from alpha motoneurons, as well as from all parasympathetic neurons and cholinergic postganglionic sympathetic neurons, it leads to muscle weakness and decreased glandular activity. Its efficacy in supporting obesity treatment is still debated, yet it is viewed as having a promising future in obesity management due to its less invasive nature, time-efficiency, and fewer side effects 12 .

The goal of this retrospective clinical study was to assess and compare efficacy of weight loss and incidence of adverse events (AEs) between botulinum toxin and semaglutide. Furthermore, the study intended to analyze and examine efficacy of additional postoperative treatments following each of these two interventions, specifically in the context of patients who had undergone laparoscopic sleeve gastrectomy (LSG) surgery.

## Patients and methods

### Inclusion and exclusion criteria

Inclusion criteria were as follows: 1) patients who were diagnosed as having simple obesity or who had undergone LSG; 2) body mass index ≥ 25 kg/m ^2^ ; 3) age range 16 to 65 years; 4) lifestyle or exercise interventions are ineffective; 5) signed an informed consent form; and 6) patients who had undergone LSG needed to have a postoperative time of more than 1 year and a BMI increase ≥ 5 kg/m ^2^ , weight gain ≥ 10 kg, increased total weight loss (TWL) ≥ 15% compared with minimum body weight, more than 20% increase in TWL compared with minimum body weight, or more than 25% increase in excess weight loss (EWL) compared with minimum weight.

Exclusion criteria were as follows: 1) gastroscopy examination and surgical contraindications, including heart failure or persistent breathing difficulties, retropharyngeal abscess, severe spinal deformity, or aortic aneurysm, inability to tolerate endoscopic examination due to physical weakness, acute stage of upper gastrointestinal corrosive inflammation or suspected upper gastrointestinal perforation, a large amount of ascites, severe abdominal distension, or severe esophageal varices; 2) pregnancy; 3) abnormal bleeding and coagulation functions; 4) a history of botulinum toxin treatment within the past 3 months; 5) a history of botulinum toxin poisoning and history of botulinum toxin allergy; 6) endoscopic detection of things such as tumors and active peptic ulcers; and 7) planned pregnancy within the past 6 months.

### Study design

This was a single-center, retrospective, open label study. We introduced the advantages and disadvantages of two treatment methods to patients at the outpatient clinic and they voluntarily decided which treatment method to adopt.

Patients were subsequently categorized into two groups—BTX and SME—based on the varying methods of intervention. Moreover, within the patient population who had undergone LSG, two additional groups were formed: LSG+BTX and LSG+SME. Body weights of these patients following LSG were chosen as baseline weights to enable direct comparison of the supplementary effects yielded by these two interventions. Following their respective surgeries, patients in each study group underwent comprehensive follow-up evaluations at 2 and 6 months postoperatively. The main outcome of this study was evaluation of the percentage of TWL and EWL. B The ideal weight for overweight (EWL=weight loss/overweight, overweight=original weight-24*height*height) was defined as a BMI of 24 kg/m ^2^ . Secondary outcomes were Gastrointestinal Quality-of-Life Index (GIQLI) and Gastroparesis Cardinal Symptom Index (GCSI).

### Ethics

Meticulous documentation of patient outcomes permitted a thorough analysis of the long-term effects and patient recovery process following the interventions. The study was approved by the Hospital Ethics Committee (approval number 2023-HX-56). The study was registered at clinicaltrials.gov.

### Endoscopic intragastric botulinum toxin-A injection

**Fig. 1:**
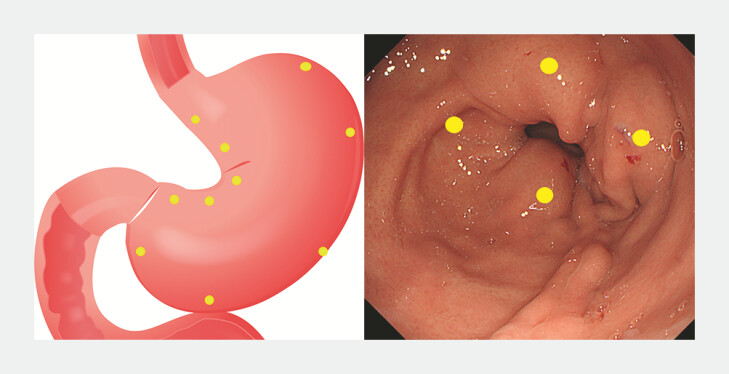
Schematic diagram and intraoperative images of endoscopic botulinum toxin injection.

The procedure involved diluting 300 units of botulinum toxin in 20 mL of 0.9% saline. Using an endoscope, a total of 20 mL of this diluted solution was then injected into the muscular layer of the gastric wall, with each of the 20 injection sites receiving 1 mL. Submucosal 1-mL botulinum toxin injection was performed ( Fig. 1 ) at four different cardinal points (3, 6, 9, and 12 o’clock) starting 2 cm from the pyloric ring and repeated four times with a distance of about 2 cm towards to incisura angularis by using an Endo Flex needle, 2.3-mm thickness, and 180 cm length (Mico-Tech, Nanjing, China). In addition, medication was administered at four points perpendicular to the gastric wall. The entire injection process was conducted within a timeframe of 10 to 20 minutes.

### Semaglutide injection

The application method for semaglutide injection (Novo Nordisk Pharma AG) is to start at 0.25 mg once a week and increase by 0.25 mg every 3 weeks until the patient can tolerate the maximum dose, with a maximum dose of ≤ 1 mg qw, for a total of 6 months. Semaglutide was stopped at 6 months.

### Lifestyle guidance

Each group of patients received dietary and lifestyle guidance from a specialized nutritionist at the beginning of treatment. A daily diet included 1000 calories and 60 minutes of exercise per day.

### Postoperative questionnaire follow-up

GIQLI scores 13 and GCSI scores 14 15 were used for postoperative follow-up of patients. The questionnaire-related content was turned into an online questionnaire and the QR code for it was sent to patients. Patients were contacted and asked to fill out the questionnaire at 2 and 6 months after surgery.

### Statistical analysis

Descriptive analyses were performed of all of study variables. Quantitative variables are expressed as medians, with minimum and maximum values, or as means with standard deviations as appropriate. Qualitative variables are expressed as absolute and relative frequencies. For the comparison of means between the two groups, student’s *t* tests were used. Statistical significance was set at *P* < 0.05. Analysis of covariance (ANCOVA) was used to statistically control for possible effects of an additional confounding variable (covariate). Data were processed with the Statistical Package for the Social Sciences, version 25.0 for Windows (SPSS Inc., Chicago, Illinois, United States). Statistical charts were prepared by GraphPad Prism10.0.

## Results

### Clinical data

**Fig. 2:**
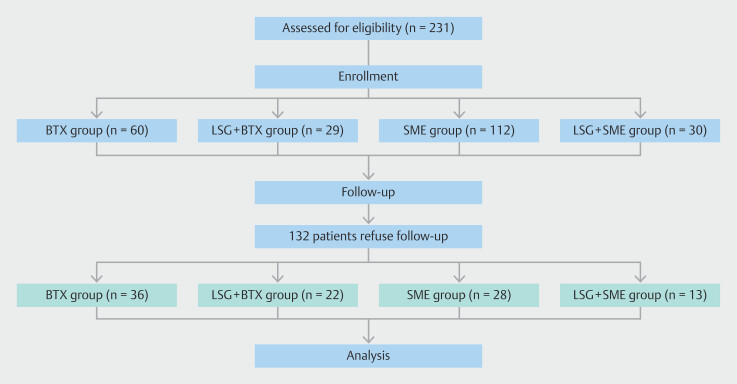
Study flow diagram.

Two hundred thirty-one patients with overweight or obesity (BMI ≥ 25) who were admitted to the Department of Metabolic Weight Loss Surgery at China-Japan Friendship Hospital from August 2022 to January 2024 were selected as the study subjects. In the end, 99 patients completed the 6-month follow-up, whereas 132 patients refused to complete the 6-month follow-up due to personal reasons. Basic information for each group of patients is shown in Table 1 . There were no statistically significant differences in gender, weight, or age composition among the patients enrolled in the four groups. Progress of the subjects during the research process is shown in the flowchart ( Fig. 2 ).

**Table:** **Table 1** Baseline characteristics of all groups.

	**BTX (n = 36)**	**LSG+BTX (n = 22)**	**SME (n = 28)**	**LSG+SME (n = 13)**	***P* value **
Age (mean ± SD)	35.75 ± 9.04	37.18 ± 8.65	33.61 ± 8.78	36.54 ± 8.81	> 0.05
Gender					> 0.05
Male	5	1	4	1	
Female	31	21	24	12	
Weight (kg) (mean ± SD)	82.91 ± 9.91	85.07 ± 10.47	87.16 ± 17.10	83.22 ± 15.36	> 0.05
BMI (kg/m2) (mean ± SD)	30.35 ± 2.96	31.62 ± 4.45	31.46 ± 3.91	30.59 ± 4.70	> 0.05
BTX, botulinum toxin; LSG, laparoscopic sleeve gastrectomy; SD, standard deviation; SME, semaglutide.					

### Weight loss

Table 2 , Table 3 , Table 4 , and Table 5 compare TWL and EWL of every group at 2 and 6 months, respectively. The BTX group lost on average 7.60% of initial weight at 2 months and 7.99% at 6 months versus 8.97% and 10.78% in the semaglutide group, respectively. The LSG+BTX group lost on average 6.01% of initial weight at 2 months and 5.62% at 6 months versus 5.46% and 8.89% in the LSG+semaglutide group, respectively.

**Table:** **Table 2** Changes at 2 and 6 months between the LSG + BTX and LSG + SME groups.

	**LSG + BTX (n = 22)**	**LSG + SME (n=13)**	***P* value **
TWL (%) (2 months) (mean ± SD)	6.01 ± 3.95	5.46 ± 4.36	0.703
TWL (%) (6 months) (mean ± SD)	5.62 ± 4.14	8.89 ± 6.15	0.069
EWL (%) (2 months) (mean ± SD)	26.65 ± 13.12	19.52 ± 23.52	0.256
EWL (%) (6 months) (mean ± SD)	24.46 ± 14.89	41.10 ± 30.56	0.085
BTX, botulinum toxin; EWL, excess weight loss; LSG, laparoscopic sleeve gastrectomy; SD, standard deviation; SME, semaglutide; TWL, total weight loss.			

**Table:** **Table 3** Changes at 2 and 6 months between the BTX and SME groups.

	**BTX (n = 36)**	**SME (n = 28)**	***P* value **
TWL (%) (2 months) (mean ± SD)	7.60 ± 6.01	8.97 ± 4.90	0.337
TWL (%) (6 months) (mean ± SD)	7.99 ± 7.65	10.78 ± 8.41	0.171
EWL (%) (2 months) (mean ± SD)	38.41 ± 32.06	51.30 ± 40.80	0.167
EWL (%) (6 months) (mean ± SD)	38.36 ± 40.50	59.35 ± 62.39	0.109
BTX, botulinum toxin; standard deviation; SME, semaglutide.			

**Table:** **Table 4** Changes at 2 and 6 months between BTX and LSG + BTX groups.

	**BTX (n = 36)**	**LSG + BTX (n = 22)**	***P* value **
TWL (%) (2 months) (mean ± SD)	7.60 ± 6.10	6.01 ± 3.95	0.233
TWL (%) (6 months) (mean ± SD)	7.99 ± 7.65	5.62 ± 4.14	0.133
EWL (%) (2 months) (mean ± SD)	38.41 ± 32.06	26.65 ± 13.12	0.057
EWL (%) (6 months) (mean ± SD)	38.36 ± 40.50	24.46 ± 14.89	0.068
BTX, botulinum toxin; EWL, excess weight loss; LSG, laparoscopic sleeve gastrectomy; SD, standard deviation; TWL, total weight loss.			

**Table:** **Table 5** Changes at 2 and 6 months between SME and LSG + SME groups.

	**SME (n = 28)**	**LSG + SME (n = 13)**	***P* value **
TWL (%) (2 months) (mean ± SD)	8.97 ± 4.90	5.46 ± 4.36	0.033
TWL (%) (6 months) (mean ± SD)	10.78 ± 8.41	8.89 ± 6.15	0.474
EWL (%) (2 months) (mean ± SD)	51.30 ± 40.80	19.52 ± 23.52	0.015
EWL (%) (6 months) (mean ± SD)	59.35 ± 62.39	41.10 ± 30.56	0.326
EWL, excess weight loss; LSG, laparoscopic sleeve gastrectomy; SD, standard deviation; TWL, total weight loss.			

From the tables, it can be seen that only the SME group and the LSG+SME group showed statistically significant differences in TWL and EWL at 2 months after surgery (the weight loss effect in the SME group was significantly better than that in the LSG+SME group). There was no statistically significant difference in postoperative weight loss effect between the other groups compared pairwise.

Table 6 and Table 7 compare nausea/vomiting (N), fullness/early satiety (F), bloating (B), and total GCSI scores in every group before and 2 and 6 months after injection.

**Table:** **Table 6** GCSI questionnaire results at baseline and 2 and 6 months of BTX and LSG + BTX groups, among the patients evaluated.

	**BTX**			***P* value **	**LSG + BTX**			***P* value **
	**Pre**	**2 months post**	**6 months post**		**Pre**	**2 months post**	**6 months post**	
**GCSI total score**	13.06 ± 4.01	13.69 ± 3.21	12.75 ± 2.92	>0.05	13.50 ± 1.92	13.09 ± 3.01	12.77 ± 3.07	> 0.05
Nausea/vomiting	4.19 ± 1.80	4.75 ± 1.48	4.53 ± 1.50	>0.05	4.82 ± 1.05	5.32 ± 2.12	5.18 ± 2.13	> 0.05
Fullness/early satiety	6.22 ± 2.58	6.22 ± 1.74	5.50 ± 1.63	>0.05	5.77 ± 1.45	6.77 ± 1.63	4.91 ± 1.34	< 0.05
Bloating	2.64 ± 1.22	2.72 ± 1.19	2.75 ± 1.30	> 0.05	2.91 ± 0.97	2.45 ± 0.80	2.50 ± 0.67	> 0.05
BTX, botulinum toxin; GCSI, Gastroparesis Cardinal Symptom Index.								

**Table:** **Table 7** GCSI questionnaire results at baseline and 2and 6 months in the SME and LSG + SME groups, among the patients evaluated.

	**SME**			***P* value **	**LSG + SME**			***P* value **
	**Pre**	**2 months post**	**6 months post**		**Pre**	**2 months post**	**6 months post**	
**GCSI total score**	10.54 ± 1.48	15.89 ± 5.53	11.14 ± 1.99	< 0.05	10.77 ± 1.23	13.54 ± 2.40	11.00 ± 1.35	< 0.05
Nausea/vomiting	3.82 ± 1.12	4.46 ± 1.55	3.64 ± 1.03	> 0.05	3.85 ± 1.21	4.62 ± 1.39	3.92 ± 0.86	> 0.05
Fullness/early satiety	4.32 ± 0.61	7.36 ± 2.87	4.75 ± 0.89	< 0.05	4.31 ± 0.48	5.77 ± 1.24	4.62 ± 0.87	< 0.05
Bloating	2.39 ± 0.74	4.07 ± 1.90	2.75 ± 1.14	< 0.05	2.62 ± 0.77	3.15 ± 1.07	2.46 ± 0.66	> 0.05
GCSI, Gastroparesis Cardinal Symptom Index; LSG, laparoscopic sleeve gastrectomy; SME, semaglutide.								

We observed no statistically significant differences in nausea/vomiting, fullness/early satiety, bloating scores, or GCSI scores following use of botulinum toxin for patients who had not undergone LSG at the three designated time points.

**Fig. 3:**
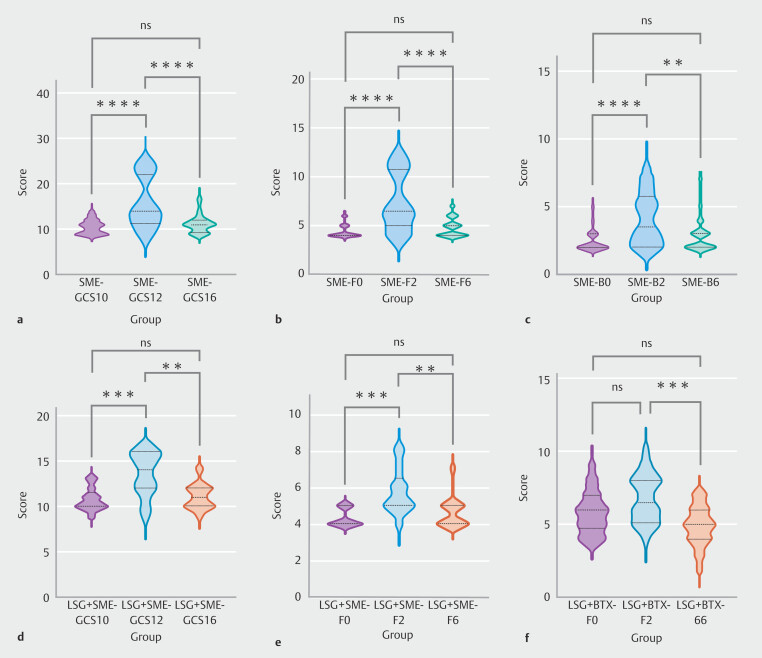
Significant intergroup differences in GCSI questionnaire results among SME. Furthermore bloating LSG+BTX groups at baseline, 2 months, and 6 months.

Conversely, when semaglutide was injected into patients who had not undergone LSG, significant statistical differences were noted in scores pertaining to fullness/early satiety, bloating, and GCSI scores at the three time points. To analyze disparities between the groups, overall ANOVA was performed first. Then the LSD test was used, and for mapping the corresponding groups, Tukey's post hoc test was used to determine differences between groups. That analysis was implemented through GraphPad Prism 10.0. In Fig. 3 **a-c** , it is evident that fullness/early satiety and GCSI scores exhibited the same particular trends. Specifically, both scores peaked 2 months post-injection and subsequently decreased significantly to levels comparable to pre-injection at 6 months (fullness/early satiety: 4.32 ± 0.61 vs 7.36 ± 2.87 vs 4.75 ± 0.89). Furthermore, bloating score mirrored the trends observed in those related to fullness/early satiety score. However, after 6 months of injections, the score decreased slightly less than fullness/early satiety group and did not regress to the level pre-injection (Bloating: 2.39 ± 0.74 vs 4.07 ± 1.90 vs 2.75 ± 1.14).

After undergoing LSG, the scores of patients who used botulinum toxin demonstrated no statistically significant differences and both remained at a high level.

After LSG, fullness/early satiety and GCSI scores in patients treated with semaglutide exhibited statistically significant differences. As depicted in Fig. 3 **d** and Fig. 3 **e** , GCSI scores in the LSG+SME group initially increased markedly, peaking 2 months post-injection, subsequently declining gradually and stabilizing at a low level (GCSI: 10.77 ± 1.23 vs 13.54 ± 2.40 vs 11.00 ± 1.35). Similarly, fullness/early satiety scores followed a comparable trend (Fullness/early satiety :4.31 ± 0.48 vs 5.77 ± 1.24 vs 4.62 ± 0.87).

Fig. 3**f** shows fullness/early satiety scores for patients who regained weight after LSG by injecting botulinum toxin for weight loss. The feeling of fullness 6 months after injection was significantly lower than 2 months after injection (6.77 ± 1.63 vs 4.91 ± 1.34).

Table 8 and Table 9 present a comparison of GIQLI scores across distinct patient groups, segmented into five core dimensions: gastrointestinal symptoms (G), emotional dimension (E), physical condition (P), social dimension (SD), and special disease conditions (SDC), at the three evaluated time points: pre-injection and 2 and 6 months post-injection.

**Table:** **Table 8** GIQLI questionnaire results at baseline and 2 and 6 months from the BTX and LSG + BTX groups, among the patients evaluated.

	**BTX**			***P* value **	**LSG + BTX**			***P* value **
	**Pre**	**2 months post**	**6 months post**		**Pre**	**2 months post**	**6 months post**	
**GIQLI total score**	125.17 ± 18.07	126.64 ± 15.24	128.134 ± 14.60	> 0.05	123.45 ± 10.78	127.05 ± 10.75	126.18 ± 8.65	> 0.05
Gastrointestinal symptoms	35.14 ± 5.60	37.75 ± 4.70	35.92 ± 5.06	> 0.05	33.86 ± 3.04	34.77 ± 3.04	35.91 ± 3.85	> 0.05
Emotional dimension	20.14 ± 5.56	20.64 ± 5.21	21.19 ± 4.81	> 0.05	20.05 ± 2.89	20.36 ± 3.47	20.09 ± 2.81	> 0.05
Physical condition	20.42 ± 3.60	21.67 ± 3.23	21.78 ± 3.23	> 0.05	20.32 ± 2.83	20.95 ± 2.75	20.41 ± 2.56	> 0.05
Social dimension	14.11 ± 2.97	14.47 ± 2.75	14.53 ± 2.34	> 0.05	13.86 ± 1.81	14.73 ± 2.21	14.55 ± 1.71	> 0.05
Special disease conditions	35.36 ± 6.12	35.11 ± 6.11	34.72 ± 7.00	> 0.05	35.36 ± 3.33	36.23 ± 2.84	35.18 ± 2.63	> 0.05
BTX, botulinum toxin; GIQLI, Gastrointestinal Quality of Life; LSG, laparoscopic sleeve gastrectomy.								

**Table:** **Table 9** GIQLI questionnaire results at baseline, 2 months, and 6 months of SME and LSG + SME group, among the patients evaluated.

	**SME**			***P* value **	**LSG + SME**			***P* value **
	**Pre**	**2 months post**	**6 months post**		**Pre**	**2 months post**	**6 months post**	
GIQLI total score	117.79 ± 20.95	130.50 ± 16.41	121.86 ± 19.65	< 0.05	122.92 ± 8.79	135.85 ± 2.97	126.62 ± 10.69	< 0.05
Gastrointestinal symptoms	29.36 ± 7.18	33.82 ± 5.06	31.79 ± 5.90	< 0.05	31.77 ± 2.98	35.38 ± 1.19	33.54 ± 1.90	< 0.05
Emotional dimension	19.61 ± 5.86	21.36 ± 5.35	19.89 ± 5.40	> 0.05	21.54 ± 1.45	23.92 ± 0.64	22.38 ± 1.56	< 0.05
Physical condition	20.50 ± 3.88	21.46 ± 3.12	20.71 ± 3.81	> 0.05	21.31 ± 1.93	22.08 ± 1.71	20.85 ± 2.38	> 0.05
Social dimension	13.57 ± 3.65	16.68 ± 4.27	13.75 ± 3.34	< 0.05	14.15 ± 1.63	16.46 ± 0.66	14.38 ± 1.66	< 0.05
Special disease conditions	34.75 ± 5.95	37.18 ± 2.74	34.79 ± 4.41	< 0.05	34.15 ± 2.85	37.92 ± 0.86	32.62 ± 2.18	< 0.05
GIQLI, Gastrointestinal Quality of Life; LSG, laparoscopic sleeve gastrectomy; SME, semaglutide.								

For patients who solely received botulinum toxin injection, without undergoing LSG, there were no statistically significant differences observed in their QoL scores. The scores consistently remained at a high level across all three timeframes.

**Fig. 4:**
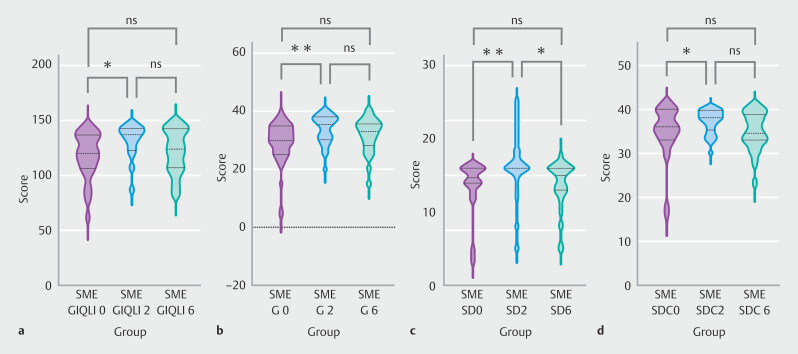
Significant intergroup differences in GIQLI questionnaire results among SME groups at baseline and 2 and 6 months.

Statistically significant differences were found in gastrointestinal symptoms, social dimension, special disease conditions, and GIQLI total score between patients receiving semaglutide injection without LSG. Fig. 4 **a-d** show a slight increase in GIQLI score 2 months post-injection, which then stabilized until 6 months, with no significant change. The gastrointestinal symptoms score followed a similar trend, with a slightly higher increase in the second month. Social dimension score initially increased then decreased, slightly exceeding baseline at 6 months. The special disease conditions score initially showed a slight increase but then remained unchanged for the subsequent 2 months following injection.

We found no significant difference in QoL scores among patients who received botulinum toxin injection following LSG. Across all three time periods evaluated, the scores remained consistently high.

**Fig. 5:**
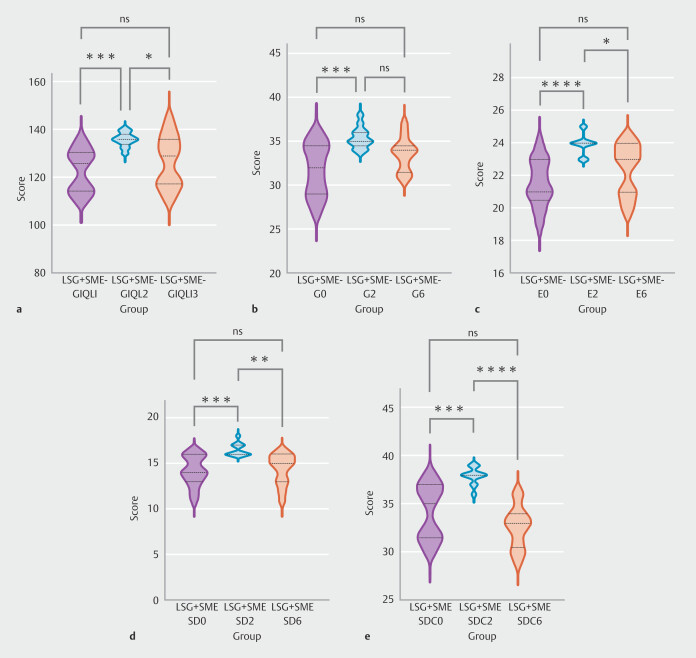
Significant intergroup differences in GIQLI questionnaire results among LSG+SME groups at baseline and 2 and 6 months.

For patients receiving semaglutide after LSG, significant differences were observed in GIQLI total score, gastrointestinal symptoms, emotional dimension, social dimension, and special disease conditions. Fig. 5 **a-e** show an initial significant increase in GIQLI score, peaking at 2 months post-injection, followed by a slight decrease but remaining above pre-injection levels. Gastrointestinal and emotional dimension scores followed similar patterns, with significant increases initially, peaking at 2 months, and then declining slightly. The social dimension score increased significantly initially but decreased significantly thereafter, ending slightly above baseline at 6 months. Special disease conditions score significantly increased after 2 months of injections but then plummeted below the initial level 6 months post-treatment.

## Discussion

As societal development progresses and living standards continue to soar, incidence of obesity has escalated markedly 16 . Among the various surgical bariatric interventions, LSG stands out as the most potent and enduring method for weight reduction. Currently, LSG is undergoing widespread clinical adoption 17 18 19 . Nevertheless, the procedure is accompanied by suboptimal weight loss or a constellation of postoperative AEs, notably nausea, vomiting, abdominal discomfort 20 , which pose significant challenges. Consequently, refining preoperative preparatory measures and postoperative adjuvant weight management strategies for LSG remains a crucial area of investigation.

At present, the main solution to the problem of weight gain after LSG surgery is to perform a second surgery to modify LSG surgery to gastric bypass surgery. This type of secondary surgery is difficult and risky, and postoperative patients may face risks such as anastomotic bleeding, anastomotic leakage, and malnutrition 21 22 .

Emerging as promising noninvasive weight loss modalities, semaglutide and botulinum toxin injections have garnered increasing attention and are progressively being incorporated into clinical practice 23 24 25 26 . There is literature indicating that multi-point injection of whole stomach botulinum toxin has a better weight loss effect than injection of botulinum toxin only in the gastric antrum. Therefore, this study used multi-point injection of whole stomach botulinum toxin as the injection method 25 . The weight loss effect of botulinum toxin in this study is comparable to previous literature 26 . Given their potential to coexist with the most prevalent bariatric surgical procedure, LSG, it is imperative to scrutinize the comparative weight loss efficacy of these therapies, both individually and in conjunction with LSG. Furthermore, assessing the impact of these combinations on patient QoL is paramount to determining the optimal pharmacological adjunct that can augment the efficacy of LSG. This comparative analysis holds significant clinical implications because it endeavors to identify the therapeutic modality that best complements LSG, thereby enhancing weight loss outcomes and optimizing patient postoperative QoLe. By elucidating the synergistic effects of semaglutide, botulinum toxin, and LSG, we can forge a more comprehensive and individualized approach to weight management, tailored to the unique needs of every patient.

At present, there is no research reporting the effect of endoscopic injection of botulinum toxin in patients with obesity after LSG. This study aimed to investigate the effect of botulinum toxin and semaglutide injection on weight loss, gastroparesis effect, and QoL in patients with simple obesity or undergoing endoscopic sleeve gastrectomy.

The study results indicate that in terms of weight loss, there is no significant difference between patients who received botulinum toxin and semaglutide injections after LSG. For patients with simple obesity, both botulinum toxin and semaglutide injections showed similar weight loss effects within a 6-month period. From the results of EWL, the findings of this study are comparable to previous research 27 .

Regarding the effect on gastroparesis, regardless of whether patients have undergone LSG, short-term effects of botulinum toxin injections are relatively less pronounced compared with semaglutide injections. This is likely due to the fact that botulinum toxin is administered as a single injection, unlike semaglutide, which is injected repeatedly over time, leading to a lesser amplitude of symptomatic response in the short term. Nevertheless, GSCI scores at the 6-month mark reveal significant reduction in symptoms of gastroparesis after botulinum toxin injection compared with semaglutide injection.

In terms of QoL, for patients with simple obesity, injection of botulinum toxin has a higher QoL score than injection of semaglutide. However, for patients who have undergone LSG, QoL was similar after injections of both drugs. In addition, regardless of whether patients had undergone LSG, QoL after semaglutide injections was found to be greatly influenced by individual differences, which may be related to development of resistance in some patients after multiple semaglutide injections, potentially leading to less pronounced effects.

Overall, during a 6-month observational period, efficacy of botulinum toxin injections was found to be comparable to that of semaglutide. Recent investigations hypothesize that botulinum toxin may diminish the satiety threshold in obese individuals by inhibiting release of ghrelin in the gastric fundus and antrum, thereby leading to a marked enhancement in postprandial satiety within the first week after a standard meal and sustaining this heightened state throughout the follow-up period 28 . Notably, botulinum toxin injections exhibit a notable advantage in terms of safety, having secured certification from the United States Food and Drug Administration. Upon analyzing various treatment modalities for obesity, intragastric administration of BTX-A was categorized as Class 1, signifying an absence of reported severe AEs 29 .

Similarly, semaglutide has demonstrated a relatively favorable safety profile in management of obesity, akin to placebo, albeit with a notably higher prevalence of gastrointestinal adverse reactions. Furthermore, it has been associated with potential side effects such as headaches, fatigue, dizziness, hypoglycemia, pancreatitis, and gallstones 30 31 32 33 . Notably, utilization of semaglutide also carries a risk of thyroid cancer, albeit the existence of an absolute correlation between semaglutide and thyroid cancer remains unclear. This potential risk should be thoroughly considered prior to and during its administration.

Given the comparable therapeutic outcomes, botulinum toxin emerges as a viable alternative for patients who exhibit allergies to or develop resistance against semaglutide. In addition, the convenience of administering botulinum toxin, requiring only a single injection as opposed to weekly injections necessitated by semaglutide, underscores its enhanced practicality. Consequently, botulinum toxin may represent a novel therapeutic option for patients suffering from severe obesity. This study has the following limitations. First, it was a retrospective, single-center study with no randomization. Patients selected their treatment, which could bias outcomes. Second only 6-month outcomes are reported. Durability of weight loss beyond 6 months is unknown. Third, the effect of semaglutide in this study is consistent with known results. Fourth, it must be acknowledged that there is still limited evidence on use of botulinum toxin in treatment of obesity, and significant differences in its weight loss effects have been observed in different studies. This variability may be due to factors such as small sample size, changes in injection site or dosage, and differences in clinical practice. Fifth, the inherent difference between a one-time BTX procedure and ongoing weekly injections makes blinding impossible and could also affect patient behavior. Sixth, this study did not have a placebo control, making it difficult to measure the true effectiveness of BTX. Seventh, the gastrointestinal symptoms reported by patients are based on their subjective feelings. In order to further elucidate the weight loss potential of botulinum toxin and evaluate its applicability as a substitute for semaglutide, rigorous randomized, double-blind, controlled trials are crucial for scientific evaluation.
